# Isothermal loop-mediated amplification (lamp) for diagnosis of contagious bovine pleuro-pneumonia

**DOI:** 10.1186/1746-6148-9-108

**Published:** 2013-05-27

**Authors:** Georg Mair, Edy M Vilei, Abel Wade, Joachim Frey, Hermann Unger

**Affiliations:** 1 Genomics Unit VetCore, University of Veterinary Medicine, Vienna, Austria; 2 Tropical Vet. Med. Laboratory, University of Veterinary Medicine, Vienna, Austria; 3 Institute of Veterinary Bacteriology, Vetsuisse, University of Bern, Bern, Switzerland; 4 LANAVET, Garoua, Cameroun; 5 Animal Production and Health Section, Joint FAO/IAEA Division of Nuclear Techniques in Food and Agriculture, Wagramer Strasse 5, P.O. Box 100, Vienna A-1400, Austria; 6 Current address: Suisselab AG, Schützenstrasse 10, Zollikofen, BE, CH-3052, Switzerland

**Keywords:** CBPP Mycoplasma mycoides, Isothermal, Loop-mediated amplification LAMP, Molecular diagnostic, Field test

## Abstract

**Background:**

Contagious Bovine Pleuropneumonia (CBPP) is the most important chronic pulmonary disease of cattle on the African continent causing severe economic losses. The disease, caused by infection with *Mycoplasma mycoides* subsp. *mycoides* is transmitted by animal contact and develops slowly into a chronic form preventing an early clinical diagnosis. Because available vaccines confer a low protection rate and short-lived immunity, the rapid diagnosis of infected animals combined with traditional curbing measures is seen as the best way to control the disease. While traditional labour-intensive bacteriological methods for the detection of *M. mycoides* subsp. *mycoides* have been replaced by molecular genetic techniques in the last two decades, these latter approaches require well-equipped laboratories and specialized personnel for the diagnosis. This is a handicap in areas where CBPP is endemic and early diagnosis is essential.

**Results:**

We present a rapid, sensitive and specific diagnostic tool for *M. mycoides* subsp. *mycoides* detection based on isothermal loop-mediated amplification (LAMP) that is applicable to field conditions. The primer set developed is highly specific and sensitive enough to diagnose clinical cases without prior cultivation of the organism. The LAMP assay detects *M. mycoides* subsp. *mycoides* DNA directly from crude samples of pulmonary/pleural fluids and serum/plasma within an hour using a simple dilution protocol. A photometric detection of LAMP products allows the real-time visualisation of the amplification curve and the application of a melting curve/re-association analysis presents a means of quality assurance based on the predetermined strand-inherent temperature profile supporting the diagnosis.

**Conclusion:**

The CBPP LAMP developed in a robust kit format can be run on a battery-driven mobile device to rapidly detect *M. mycoides* subsp. *mycoides* infections from clinical or post mortem samples. The stringent innate quality control allows a conclusive on-site diagnosis of CBPP such as during farm or slaughter house inspections.

## Background

Contagious Bovine Pleuropneumonia (CBPP) caused by *Mycoplasma mycoides* subsp. *mycoides* (previously further specified as Small Colony (SC) type) (Manso-Silván et al. [1]) is considered as the main plague of cattle in Africa since the eradication of rinderpest (Roeder [2]). In recent years, CBPP re-emerged from where it had persisted and has spread to areas from which it had previously been eradicated, thus stimulating new, large-scale efforts to contain or possibly eradicate epidemics (http://www.fao.org). The pathogen is considered highly contagious and often there is only a slow development of clinical signs. Current vaccines against CBPP confer only partial and short-lived protection and there is some concern regarding their biosafety (Mbulu et al. [3]; Thiaucourt et al. [4]; Wesonga et al. [5]). Hence, rapid and reliable diagnosis of CBPP combined with traditional control measures including reduction of animal movement and isolation or stamping-out of affected animals, remains the most efficient way to control the disease. Serological methods are generally used for determining the CBPP status at herd level (Amanfu et al. [6]; Bruderer et al. [7]; Le Goff and Thiaucourt [8]; Muuka et al. [9]; Schubert et al. [10]; Tardy et al. [11]). Bacterial isolation is still the reference method for detection of the pathogen. However, bacterial cultivation of mycoplasma species in general and subsequent identification is cumbersome due to the fastidious growth requirements of these bacteria.

During the last two decades, several conventional and real-time PCR methods (qPCR) for rapid diagnosis of CBPP and early outbreak warning have been developed (Bashirrudin et al. [12]; Fitzmaurice et al. [13]; Lorenzon et al. [14]; Miserez et al. [15]; Schnee et al. [16]; Vilei and Frey [17]). Although such methods are specific and sensitive they depend on a laboratory infrastructure with sophisticated equipment and highly trained personnel. Most PCR protocols for CBPP diagnosis rely on the samples being pre-enriched for mycoplasmas and/or require expensive and laborious extraction methods. Such methods are not generally available in the rural areas of Africa where CBPP is possibly endemic and rapid diagnosis of the disease is essential. Another issue is that transportation of infected samples over long distances drastically affects the viability of the bacteria rendering them unfit for culturing and/or resulting in spoilage of the nucleic acid*.* To address the need for an in-the-field diagnostic method for CBPP, an isothermal loop-mediated amplification (LAMP) has been developed (Notomi et al. [18]). This is based on autocycling DNA synthesis under isothermal conditions in the presence of a thermophilic strand-displacing DNA polymerase that contains the 5’ → 3’ polymerase activity but lacks 5’ → 3’ exonuclease activity (such as Bst or Bsm polymerase). The technique uses four specific primers recognizing six distinct regions on the DNA template (Notomi et al. [18]). The CBPP LAMP developed is fulfilling most of the criteria for an Affordable, Sensitive, Specific, User-friendly, Robust and rapid, Equipment free and Deliverable to the end user (ASSURED) diagnostic system (Mabey et al. [19]).

## Methods

### Mycoplasma strains and DNA extraction

In this study, 83 strains of mycoplasmas representing 17 different species or subspecies including 28 isolates of *M. mycoides* subsp. *mycoides* were used (Additional file 1: Table S1). Mycoplasmas were grown in a standard medium (Axcell Biotechnologies, St. Genis l’Argentière, France) at 37°C to a density of 10^8^–10^9^ colony-forming units (CFU) per ml or on solid mycoplasma agar medium (Axcell Biotechnologies). Handling of live *M. mycoides* subsp. *mycoides* was performed in a biological safety laboratory fulfilling the BL3 containment safety standards. Lysis of mycoplasmas with GES buffer (5 M guanidium thiocyanate, 100 mM EDTA, 0.5% *N*-lauroylsarcosine) and extraction of genomic DNA were performed as previously described (Pilo et al. [20]).

### Clinical samples

To allow for the use of the LAMP for diagnosis in the field, the amplification should effectively work without the need to extract DNA. This was studied using clinical samples of pleural fluid and bronchial lavage. Sequential bronchial lavage fluids were collected from cattle during an earlier experimental infection trial (Miserez et al. [15]; ethical approval by Swiss Federal Ministry of Veterinary Affairs). In addition, simulated clinical samples consisting of *M. mycoides* subsp. *mycoides* suspension supplemented with blood or squeezed liver tissues were prepared. The samples from inoculated cattle from Cameroun were from a vaccination trial (ethically approval by the board of directors of LANAVET, Cameroun; 2010).

### Sample preparation

Samples (1.5 μl) were added to 25 μl of LAMP buffer (66 mM Tris-HCl pH 8.8, 32 mM KCl, 32 mM (NH_4_)_2_SO_4_, 16 mM MgCl_2_, 0.3% (v/v) Tween 20) and heated for five minutes at 95°C (a process available in the reading device), followed by immediate chilling and pulse centrifugation to sediment precipitates. Chilling was necessary before addition to the LAMP master mix to avoid damaging the strand displacement polymerase.

### LAMP primer design

The gene sequence encoding a putative pro-lipoprotein of *M. mycoides* subsp. *mycoides*, locus tag MSC_0500 (GenBank accession number: BX293980.2 from the type strain PG1; Westberg et al. [21]) was selected for primer design. Since the initial primer design with PrimerExplorer 3.0 (Eicken, Japan; http://www.primerexplorer.jp/elamp3.0.0/index.html) did not yield a performing primer set (high GC content), we first designed the outer primers Msc3.1-F3 and Msc3.1-B3 (Table 1) with Primer Express 2.0 (Life Technologies, Grand Island NY, USA) according to the heat map profile. These colder sequences were then applied to PrimerExplorer 3.0 for the design of the inner primers Msc3.1-FIP and Msc3.1-BIP (Table 1). The melting temperatures (Tm) of the resulting primer sequences were all in the range of 56°C. Primers were then tested for hairpin structures, extensible primer hybrids and specificity by NetPrimer (PREMIER Biosoft, USA; http://www.premierbiosoft.com/netprimer/index.html) and Primer-BLAST (http://www.ncbi.nlm.nih.gov/tools/primer-blast/).

**Table 1:** Nucleotide sequences of the CBPP LAMP primers used

Msc3.1-F3	ACTACTTGTTGTTGTAGTGTTTG
Msc3.1-B3	ATGGTGGTTTATCAAATGATGA
Msc3.1-FIP	TCAAAGAGATAATTCTACTTCAGCTAAGGATCCTGAAGAACCAGAA
Msc3.1-BIP	TCTAGCAGAATTTTTTGCACTTAACAAAGTTGATAACGCAATAGAGC

### LAMP reaction conditions

The total volume of the LAMP assays was 25 μl i.e. 15 μl of LAMP buffer including the sample and 10 μl of master mix for final concentrations of 460 mM trehalose (alternative enhancer), 0.4 mM dNTP, 1.25 μM of each of the inner primers (Msc3.1-FIP and Msc3.1-BIP) and 0.25 μM of each of the outer primers (Msc3.1-F3 and Msc3.1-B3). The reaction mix also contained 8 U Bsm polymerase (Fermentas Thermo Scientific, St. Leon-Rot, Germany) and 0.4 × EvaGreen® dye (20×; Biotinum Inc., Hayward, CA, USA). Standard reactions were run for 60 min at 58°C in an ESEQuant Tube Scanner TS95 (Qiagen, Hilden, Germany) recording fluorescence from DNA bound EvaGreen once per minute. The optimal reaction temperature was predetermined on a gradient real-time cycler (StepOnePlus™; Life Technologies, Grand Island NY, US).

### Quality assurance

To allow for a quality assurance protocol, the standard procedure for real-time PCR, melting curve analysis (MCA) and an alternatively re-association curve analysis was tested on a real-time platform (Rotor-Gene Q, Qiagen, Hilden Germany). After the LAMP process, for MCA samples were heated from 65°C to 90°C in 1°C/min steps with consecutive measurement at each step. For re-association of melted DNA (amplicons), reactions were cooled down from 90°C to 65°C in the same fashion. The amplicon inherent Tm was assessed with Oligo Calc (http://www.basic.northwestern.edu/biotools/oligocalc.html) and POLAND (http://www.biophys.uni-duesseldorf.de/html/local/POLAND/poland.html).

### LAMP result evaluation

LAMP is a continuous process, unlike PCR, with parallel amplification and strand separation. Thus, only a “quasi” exponential function can be expected in short time increments and only the measurement of fluorescence in intervals of 1 minute or longer presents a continuous sigmoidal curve. For this exercise, we chose 1 min increments to evaluate the curve and defined an increase of > 30 mV/minute as a significant increase (Additional file 1). This algorithm was implemented in the Tube Scanner software and has the following limiting factors: the baseline starts after the third measurement and ends after the seventh, whereas the mean of the mV values represents the baseline value (Bv). The curve is classified as positive if the increase of signal (slope) above the mean of the baseline is > 30 mV for a minimum of 3 measurements (Additional file 1). The absolute time-point of detection Td (quantification) is determined when the slope increase is > 30 mV for the first time at a tube scanner excitation intensity of 36%.

## Results

### Evaluation of analytical sensitivity and specificity

To evaluate the specificity of the CBPP LAMP reaction, templates of purified DNA samples corresponding to approximately 10^6^ genome equivalents or suspensions of mycoplasmal cultures corresponding to approximately 10^6^ cells from 28 different strains of *M. mycoides* subsp. *mycoides* and from 57 strains of 15 other related mycoplasma species or subspecies (Additional file 1: Table S1) were examined in the CBPP LAMP. The CBPP LAMP gave positive signals for all 28 strains of *M. mycoides* subsp. *mycoides*, but not for any of the other 57 mycoplasma species. The LAMP reactions for the negative control remained negative, even when the reactions extended to 90 minutes.

**Figure 1:**
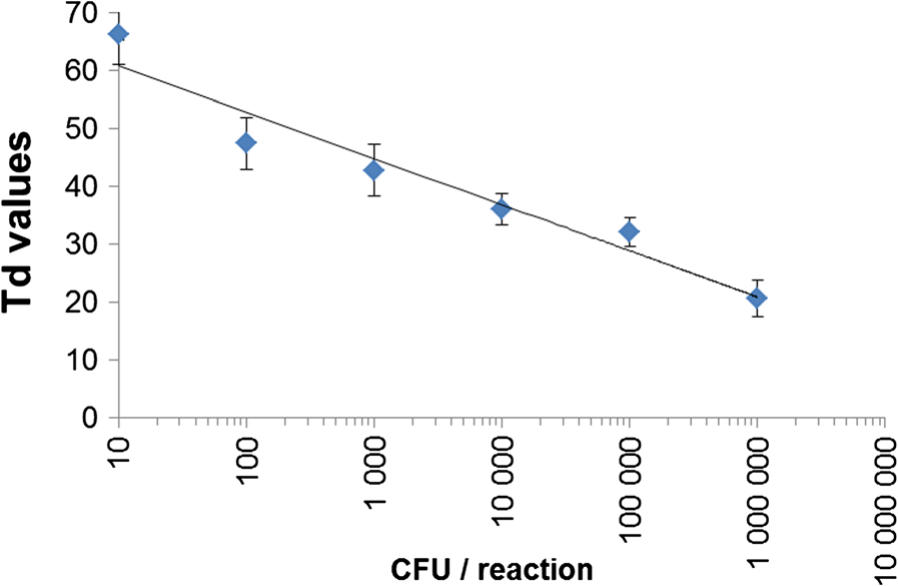
**Sensitivity of the CBPP LAMP: Titration curve based on diluted culture samples of *M. mycoides *subsp. *mycoides *strain Afadé directly employed in the reaction.**

To assess the limit of detection, a series of 10 fold dilutions of a suspension culture of *M. mycoides* subsp. *mycoides* strain Afadé were used as template. As shown in Figure 1, the method detects 1000 cells per reaction at a Time of detection (Td) of 45 minutes, while the detection of 10 cells requires an extended analysis time of Td = 67 minutes.

### Quality assurance

**Figure 2:**
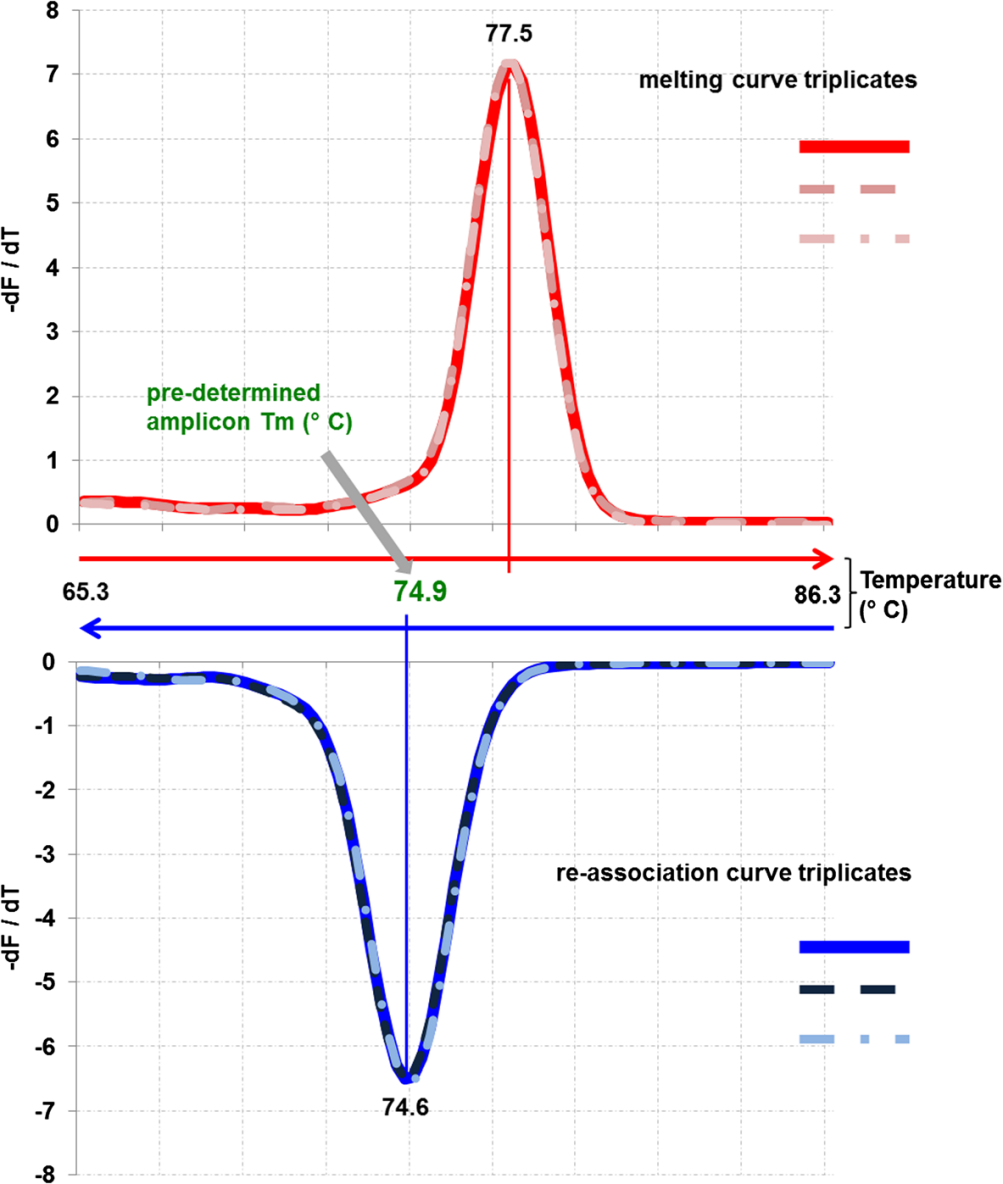
**Melting curve and re-association curve analysis of *M. mycoides *subsp. *mycoides *type strain PG1; directly from broth culture.** The pre-determined Tm (74.9°C) corresponds to the value achieved through re-association (74.6°C) of exo-thermally separated LAMP products.

For pathogen diagnosis, a quality assurance protocol is necessary. We performed MCA, the standard for qPCR and compared the result with the Tm for our CBPP amplicon calculated using two methods (nearest neighbour and Poland algorithm, respectively; Breslauer et al. [22]; Steger [23]). Interestingly, the calculated Tm (74.9°C) did not correspond to the values measured (78.4 to 79.0°C, dependent on the sample origin) (Figure 2). The analysis of the inverse protocol, re-association curve analysis (RCA) gave values matching the calculated ones thus allowing a reliable QA for CBPP LAMP diagnosis.

### Analysis of clinical samples

**Figure 3:**
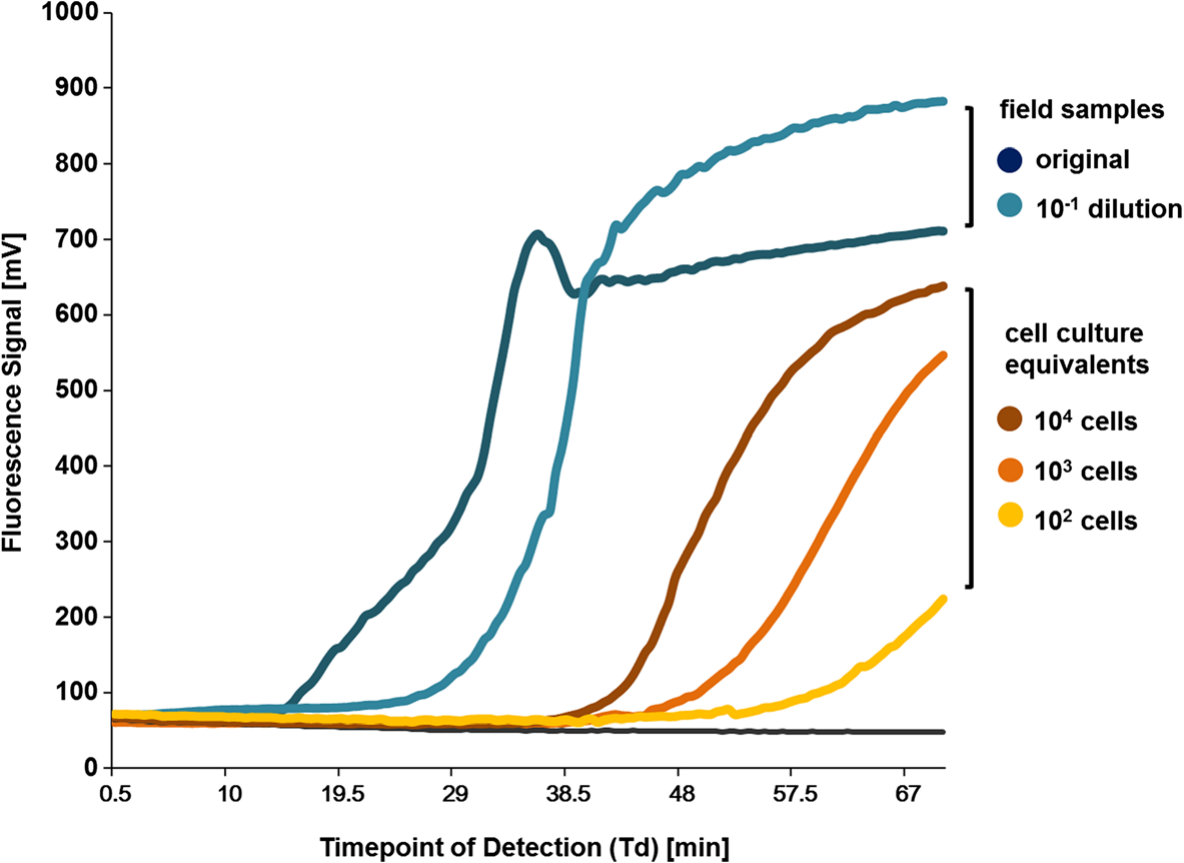
**Calibration of the CBPP LAMP.** Upper panel: Fluorometric output of LAMP amplifications using non-processed pleural fluid samples from a CBPP infected cow. Lower panel: Titration of *M. mycoides* subsp. *mycoides* strain Afadé culture fluid at various concentrations.

The assessment of the test performance for the crude samples gave for the spiked samples a Td of around 45 minutes (10^3^*Mmm*/reaction). Pleural fluid samples from two cows, with bacteriologically confirmed CBPP taken at necropsy, resulted in Td values ranging between 20 and 35 minutes (Figure 3).

LAMP analyses of bronchial lavage samples taken sequentially from in-contact animals (Miserez et al. [15]) gave very similar results to the highly sensitive nested PCR based on P72 gene encoding the lipoprotein LppA (Tables 2, 3), showing that the CBPP LAMP was suitable for both clinical samples from necropsy and from live animals. The AFADE 16 samples gave identical results. The LAMP was additionally positive 4 times and the n-PCR was positive once more. For the L2 strain, 27 samples corresponded and additionally 5 times only LAMP was positive.

**Table 2:** **Detection of *M. mycoides *subsp*. mycoides *in bronchial lavage fluid of cattle that were experimentally infected by contact with animals that were directly infected 4 weeks before with *M. mycoides *subsp*. mycoides *a) strain Afadé [**[15]**] or b) strain L2 [**[15]**]**

**Days before/after contact infection**	**Viable *M. mycoides *subsp. *mycoides */ ml**	Nested PCR*	**LAMP**
- 28		**-**	**-**
- 7		**-**	**-**
+ 13	1.3 × 10^4^	**+**	**+**
+ 21	4.0 × 10^4^	**+**	**+**
+ 28	3.5 × 10^3^	**+**	**+**
+ 35	2.5 × 10^3^	**+**	**+**
+ 42	7.0 × 10^7^	**+**	**+**
+ 49	5.5 × 10^6^	**+**	**+**
+ 56	1.8 × 10^7^	**+**	**+**
+ 63	7.0 × 10^8^	**+**	**+**
+ 70		**-**	**+**
+ 77		**-**	**-**
+ 84		**-**	**-**
+ 92		**-**	**+**
+ 98		**-**	**-**
+ 105	1.0 × 10^2^	**+**	**+**
+ 112		**-**	**+**
+ 119		**-**	**+**
+ 126		**+**	**-**
+ 134		**-**	**+**
+ 141		**-**	**-**
+ 147		**-**	**-**

* nested PCR results [15].

**Table 3:** **Detection of *M. mycoides *subsp*. mycoides *in bronchial lavage fluid of cattle that were experimentally infected by contact with animals that were directly infected 4 weeks before with *M. mycoides *subsp*. mycoides *a) strain Afadé or b) strain L2 (Miserez et al., [**[15]**])**

**Days before/after contact infection**	**Viable *M. mycoides *subsp. *mycoides */ ml**	Nested PCR*	**LAMP**
- 21		**-**	**-**
+ 16		**-**	**-**
+ 21		**-**	**-**
+ 29		**-**	**-**
+ 35		**-**	**-**
+ 42		**-**	**-**
+ 49		**-**	**-**
+ 56		**-**	**-**
+ 64		**-**	**-**
+ 70		**-**	**+**
+ 77	1.0 × 10^2^	**+**	**+**
+ 84		**+**	**+**
+ 92	2.5 × 10^2^	**+**	**+**
+ 100	3.5 × 10^2^	**+**	**+**
+ 105	8.5 × 10^2^	**+**	**+**
+ 112	1.0 × 10^2^	**+**	**+**
+ 119	1.0 × 10^2^	**+**	**+**
+ 125	1.0 × 10^2^	**+**	**+**
+ 132		**+**	**+**
+ 140		**-**	**+**
+ 146		**-**	**+**
+ 153		**+**	**+**
+ 160		**+**	**+**
+ 168		**-**	**+**
+ 175		**+**	**+**
+ 181		**+**	**+**
+ 188	1.0 × 10^2^	**+**	**+**
+ 195	1.0 × 10^2^	**+**	**+**
+ 202		**-**	**-**
+ 209		**-**	**-**
+ 216		**-**	**+**
+ 223		**-**	**-**

* nested PCR results [15].

Samples collected from a challenge trial in Cameroun revealed that not only were pulmonary samples and pleural fluids suitable for *Mmm* detection by LAMP, but the serum also gave valid results (Table 4).

**Table 4:** **Experimental samples from Cameroun tested for *M. mycoides *subsp. *mycoides *(Mmm) by different methods and LAMP; all samples were collected >45 days post intubation and showed specific pathology on post mortem PM except 8410**

**Origin**	**Specimen ID**	**PCR**	**cELISA**	**Culture**	**LAMP**
Mmm inoculated cow, 8429	Serum	nd	**+**	nd	**+**
	Pleural fluid	**+**	nd	**+**	**+**
Mmm inoculated cow, 8462	Serum	nd	**+**	nd	**+**
	Pleural fluid	**+**	nd	**+**	**+**
Mmm inoculated cow, 05	Serum	nd	**+**	nd	**+**
	Lung	**+**	nd	**+**	**+**
	Pleural fluid	**+**	nd	**+**	**+**
Bov. before inoculation; 8178	Serum	*-*	nd	nd	*-*
Mmm inoculated cow, 8178	Serum	nd	**+**	nd	**+**
Mmm inoculated cow, 07	Lung	**+**	nd	**+**	**+**
	Pleural fluid	**+**	nd	**+**	**+**
Mmm inoculated cow, 08	Lung	**+**	nd	**+**	**+**
	Pleural fluid	**+**	nd	**+**	**+**
Mmm inoculated cow, 8454	Serum	nd	**+**	nd	**+**
	Serum	nd	**+**	nd	**+**
Mmm inoculated cow, 49	Lung	**+**	nd	**+**	**+**
Mmm inoculated cow, 50	Lung	**+**	nd	**+**	**+**
Mmm inoculated cow, 56	Pleural fluid	**+**	nd	**+**	**+**
Mmm inoculated cow, 8410	Lung	*-*	nd	nd	*-*
	Pulmon. lymph node	*-*	nd	nd	*-*

*nd*: not determined.

## Discussion and conclusion

Sensitive, reliable and time-saving molecular diagnostic tests, vital for the successful control of CBPP, mostly rely on TaqMan®-based real-time PCR for the detection of the pathogen *M. mycoides* subsp. *mycoides* in extracted and often pre-cultured clinical samples (Gorton et al. [24]; Schnee et al. [16]; Vilei and Frey [17]). Whilst these are broadly accepted as the most specific and sensitive method and as reference tests in control and surveillance programs, they depend on an elaborate and expensive laboratory infrastructure; sophisticated equipment and personnel with advanced molecular diagnostic knowledge hence, are not applicable to field conditions where CBPP is endemic and where on-site diagnosis is vital to provide confirmation of disease. A new and highly efficient isothermal amplification protocol for the detection of CBPP was developed that offers the same advantages of specificity and sensitivity of the real-time PCR methodology, but does not need the sophisticated laboratory-based equipment and is thus applicable to field conditions. This was accomplished through the design of a specific LAMP primer set for an already optimized master mix. To make the test suitable for the field, the master and the primer mix were lyophilized and are thus thermostable for a longer period (>1 year) and when rehydrated for at least one week on the bench. Additionally, the ready mixes allow the test to be carried out in only two pipetting steps (sample dilution and master mix assembly) thus avoiding errors and reducing training needs of operators.

Because the sample preparation and the amplification steps are run in a battery-driven instrument, the test can easily be performed at any location. The continuous reading of the fluorescence during amplification allows a real-time analysis of the results by the introduction of a minimum absolute increase value for positivity thus avoiding background problems usually faced with threshold values. The interpretation of results simply consists of monitoring the fluorescence increase of more than 30 mV for a minimum of two consecutive readings, which corresponds to a positive sample. Furthermore, these evaluation parameters can be programmed and therefore only the results are displayed on the instrument.

A QA procedure was established corresponding to the published Tm prediction models (Steger [23]; Schütz and van Ahsen [25]; Kibbe [26]). Despite the wide application of melting curve analysis (MCA), the re-association analysis in our setup gives the advantage of a direct correspondence to the established prediction calculations (Breslauer et al. [22]; Steger [23]) and does not require integration of surrogate parameters such as enhancers or dye factors. To perform the re-association analysis, specific software for the tube scanner (ESEmelt) was developed together with the producer based on the Savitzky-Golay filter algorithm (Savitzky and Golay [27]). False positive results due to random amplification of non-specific target DNA or contamination were excluded through the re-association analysis carried directly after amplification step without opening the vessel.

The analytical sensitivity (titration) of our test was comparable to the diagnostic sensitivity (clinical samples) better than, the real-time PCR or nested PCR. As in real-time PCR, heat-inactivated samples, after amplification, are kept in the closed tubes to avoid cross-contamination and to facilitate further analysis in a laboratory.

Regarding the ASSURED parameters published by Mabey the test fulfils all criteria with the exception of freedom from instrumentation. We believe that for a quality assured field result quantitative parameters which can be transmitted instantly to a reference laboratory are important thus allowing an immediate response which might be rather expensive.

The developed CBPP LAMP is an ideal, simple, robust and precise method to diagnose *M. mycoides* subsp. *mycoides* in an outbreak situation or during regular slaughterhouse inspections in monitoring programs for CBPP prevalence assessment.

## Competing interests

The authors declare that they have no competing interests.

## Authors’ contributions

HU and GM contributed equally to the study design and in drafting the manuscript. GM took the lead on the assay development and initial testing. JF designed the evaluation procedure and EMV performed the evaluation of the test performance. AW carried out the field evaluation. GM and HU developed the result evaluation. JF and EMV provided critical feedback on the manuscript. All authors read and approved the final manuscript.

## Supplementary Material

Additional file 1 Supplement. Click here for file
