# Supplementary material for: Isothermal loop-mediated amplification (lamp) for diagnosis of contagious bovine pleuro-pneumonia
Source: BMC Vet Res. 2013 May 27;9:108. doi: 10.1186/1746-6148-9-108 (PMC3671963; doi:10.1186/1746-6148-9-108)
Supplement: Additional file 1 — Supplement. [file 1746-6148-9-108-S1.docx]

**Supplement**

The evaluation of a fluorometric LAMP employing neat samples needs a careful approach. The sample will inherently change the matrix of the reaction and can add even some color. Applying a relative measurement is prone with error due to the individual influence of the matrix affects. Thus, an absolute measure of fluorescence was necessary. As the photo multiplier in the instrument already generates a current, this can be expressed in mV.

For a given curve of absolute measurement values:


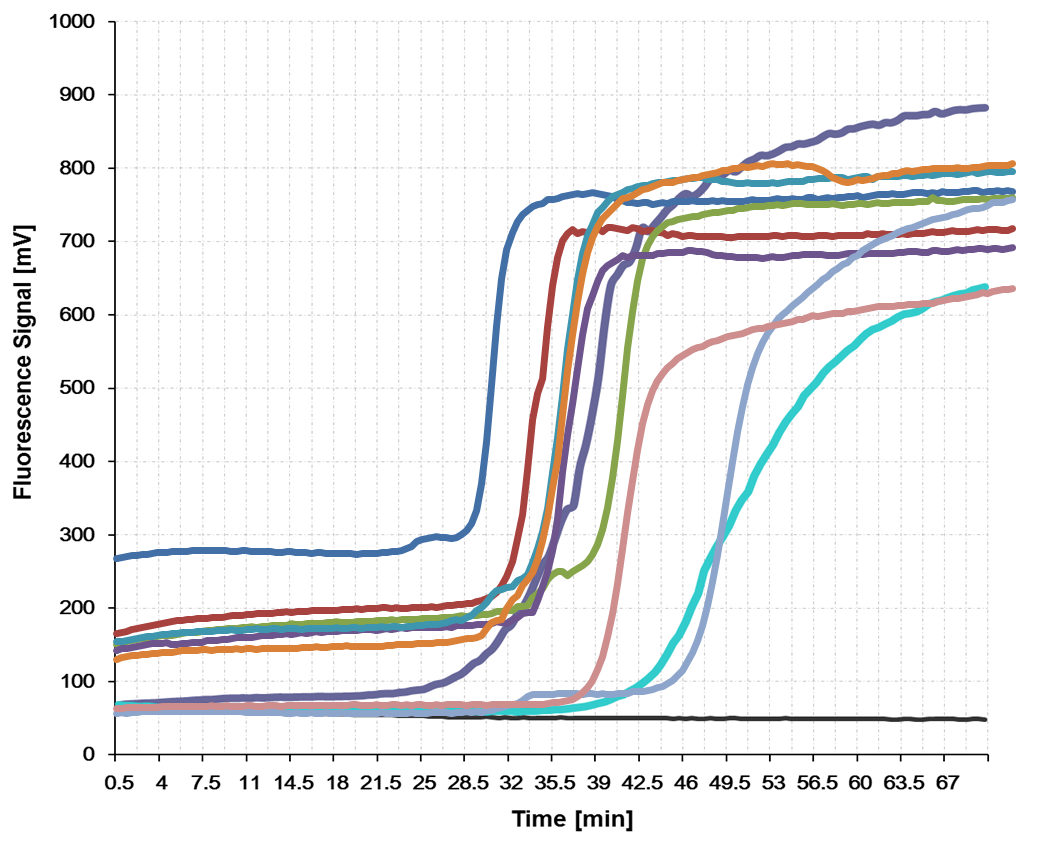


1. First of all, a baseline calling is performed to determine a general threshold;
2. Then, the increase of the slope has to be determined.


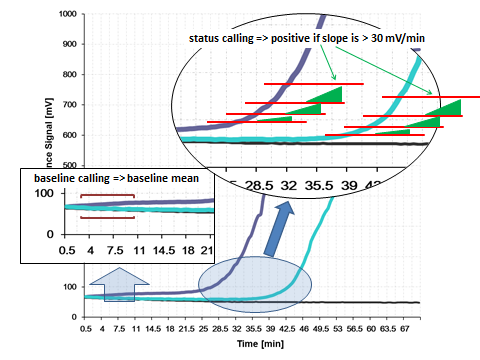


The fluorescence slope increases over time as the amplification increases in its efficiency (longer amplicons through extension). Thus, the reading presents an ever increasing curve. A value of > 30 mV/ min indicated a positive result as evaluated with reference samples. The tube scanner software can be programmed to analyze the samples accordingly.

To quantify a LAMP result is only possible with the same matrix background and of course the identical strain/ pathogen. For the quantification we applied the following procedure:

After determination of the baseline mean of 4 minutes/readings, the time point of detection Td was chosen to be the second time point with the measurement value being 30 mV above the threshold.

Table 1: Mycoplasma strains used for specificity testing and respective results

| **Species** | **Strain** | **Origin** | **Isolated** | **Host** | **Template** | | **LAMP**  **CBPP** |
| --- | --- | --- | --- | --- | --- | --- | --- |
|  |  |  |  |  | DNA | culture |  |
| *Mycoplasma mycoides* subsp*. mycoides* | PG1 | Africa | 1931 | Cattle/ [NCTC 10114^T^] | X |  | + |
|  | 2059 | Spain | 1984 | Cattle/lung | X |  | + |
|  | B773/125 | Portugal | 1991 | Cattle/semen | X |  | + |
|  | C305 | Portugal | 1993 | Goat/lung | X |  | + |
|  | O326 | Portugal | 1993 | Sheep/milk | X |  | + |
|  | PO 1967 | France | 1967 | Cattle/lung | X |  | + |
|  | PO 2 | France | 1980 | Cattle/lung | X |  | + |
|  | 2022 | France | 1984 | Cattle/lung | X |  | + |
|  | L2 | Italy | 1993 | Cattle/lung | X |  | + |
|  | 402 | Italy | 1990 | Cattle/lung | X |  | + |
|  | 6479 | Italy | 1992 | Cattle/lung | X |  | + |
|  | Afadé | Cameroon | 1968 | Cattle/lung | X |  | + |
|  | B237 | Kenya | 1987 | Cattle/lung | X |  | + |
|  | Filfili | Senegal | 1965 | Cattle | X |  | + |
|  | Fatick | Senegal | 1968 | Cattle | X |  | + |
|  | B17 | Chad | 1967 | Zebu | X |  | + |
|  | 9050-529/1 | Ivory Coast | 1990 | Cattle | X |  | + |
|  | 91130 | CAR | 1991 | Cattle | X |  | + |
|  | 94111 | Rwanda | 1994 | Cattle | X |  | + |
|  | 95014 | Tanzania | 1995 | Cattle | X |  | + |
|  | T1/44 | Tanzania | 1952 | Cattle/vaccine strain | X |  | + |
|  | T1*lppQ*^–^RSP1 | recombinant mutant | 2008 | Transposon-based random mutagenesis |  | X | + |
|  | T1/Sr50 | Tanzania | 1952 | Cattle/vaccine strain | X |  | + |
|  | KH_3_J | Sudan | 1940 | Cattle/vaccine strain | X |  | + |
|  | Gladysdale | Australia | 1953 | Cattle | X |  | + |
|  | DVZ | Australia | 1965 | Cattle | X |  | + |
|  | R575 | Australia | 1965 | Cattle | X |  | + |
|  | V5 | Australia | 1936 | Cattle/vaccine strain | X |  | + |
| *Mycoplasma leachii* | PG50 | Australia | 1963 | Cattle [NCTC 10133^T^] | X |  | - |
|  | 99/014/6 | Australia | 1999 | Cattle/joint | X |  | - |
|  | CP291 | Portugal |  | Goat/lung | X |  | - |
|  | PAD3186 | India | 1993 | Goat/milk | X |  | - |
|  | FRD424 | India | 1993 | Goat/milk | X |  | - |
|  | Calf 1 | Nigeria | <1990 | Cattle/blood | X |  | - |
|  | D318b | Germany | <1990 | Cattle/semen | X |  | - |
|  | C2306 | Portugal | <1990 | Cattle | X |  | - |
|  | D424 | Germany | <1990 | Cattle/preputium | X |  | - |
|  | QR1/92 | Australia |  |  | X |  | - |
|  | 4055 | France | 1991 | Cattle/lung | X |  | - |
|  | B144P | USA | 1956 | Cattle/joint | X |  | - |
| *Mycoplasma mycoides* subsp*. capri* | PG3 | Turkey | 1950 | Goat/type strain [NCTC 10137] | X |  | - |
|  | N/108 | Benin | 1950 | Goat/lung | X |  | - |
|  | capri L | France | 1975 | Goat | X |  | - |
|  | 9139/11-91 | Turkey | 1991 |  | X |  | - |
|  | WK354/80 | Switzerland | 1980 | Goat | X |  | - |
|  | 213 | India | 1984 | Goat |  | X | - |
|  | Y-goat | Australia | 1956 | Goat/type strain (serovar *Mmm*LC) | X |  | - |
|  | 152/93 | Grand Canary | 1993 | Goat | X |  | - |
|  | LC8065 | France |  | Goat | X |  | - |
|  | D2482/91 | Switzerland | 1991 | Goat | X |  | - |
|  | 950010 | France | 1995 | Goat |  | X | - |
|  | D2083/91 | Switzerland | 1991 | Goat | X |  | - |
|  | CP271 | Portugal | 1991 | Goat | X |  | - |
|  | D2503 | Switzerland | 1991 | Goat | X |  | - |
| *Mycoplasma capricolum* subsp*. capricolum* | California kid | USA | 1955 | Goat/type strain [NCTC 10154] | X |  | - |
|  | 173/87 | Greece | 1987 | Sheep | X |  | - |
|  | 6443.90 | France | 1990 | Goat |  | X | - |
| *Mycoplasma capricolum* subsp*. capripneumoniae* | F38 | Kenya | 1976 | Goat/lung/type strain [NCTC 10192] | X |  | - |
|  | 9081-487p | Oman | 1990 | Goat | X |  | - |
|  | Gabès | Tunisia | 1981 | Goat | X |  | - |
| *Mycoplasma* *feriruminatoris* | 321/93 | Germany | 1993 | Alpine ibex/joint | X |  | - |
| *Mycoplasma bovis* | PG45 | USA | 1962 | Cattle/udder/type strain [NCTC 10131] | X |  | - |
|  | ML1 | France | pre-1999 | Rabbit/lung | X |  | - |
|  | 221/89 | Germany | 1980-1990 | Cattle/milk | X |  | - |
|  | 86p | Belgium | 1990-2000 | Cattle/milk | X |  | - |
|  | 39G | Belgium | 1990-2000 | Cattle/bronchoalveolar lavage fluid | X |  | - |
|  | 0435 | Belgium | 1990-2000 | Cattle/bronchoalveolar lavage fluid | X |  | - |
|  | 9585 | Belgium | 1990-2000 | Cattle/bronchoalveolar lavage fluid | X |  | - |
|  | 2610 | UK | 1990-2000 | Cattle/joint fluid | X |  | - |
| *Mycoplasma bovirhinis* | PG43 |  | 1967 | Cattle/respiratory tract/type strain [NCTC 10118] | X |  | - |
|  | O/D1467 | Switzerland | 2007 | Cattle/bronchus | X |  | - |
| *Mycoplasma bovigenitalium* | PG11 | United Kingdom |  | Cattle/genital tract/type strain [NCTC 10122] | X |  | - |
|  | 2D | Australia | 1974 | Sheep (former sero group 11 strain) | X |  | - |
| *Mycoplasma agalactiae* | PG2 | Spain | 1952 | Goat/type strain [NCTC 10123] | X |  | - |
|  | 3990 | France |  |  | X |  | - |
|  | 5725 | France | 1990 | Sheep | X |  | - |
| *Mycoplasma putrefaciens* | KS1 | USA |  | Goat/type strain [NCTC 10155] | X |  | - |
| *Mycoplasma conjunctivae* | HRC/581 | USA | 1972 | Sheep/eye/type strain [NCTC 10147] | X |  | - |
| *Mycoplasma hyopneumoniae* | J |  | 1965 | Swine/type strain [NCTC 10110] | X |  | - |
| *Mycoplasma bovoculi* | M165/69 | Canada | 1969 | Cattle/eye/type strain [NCTC 10141] |  | X | - |
| *Mycoplasma ovipneumoniae* | Y98 | Australia | 1972 | Sheep/respiratory tract/type strain [NCTC 10151] |  | X | - |
| *Mycoplasma arginini* | G230 | USA | 1968 | Mouse/brain/type strain [NCTC 10129] | X |  | - |
| *Mycoplasma pneumoniae* | FH | USA | 1944 | Human/lung/type strain [NCTC 10119] | X |  | - |
